# The fMRI study for acupuncture on shift work sleep disorder: Study protocol for a randomized controlled neuroimaging trial

**DOI:** 10.1097/MD.0000000000022068

**Published:** 2020-09-04

**Authors:** Yanzhe Ning, Xueyan Liu, Hao Yao, Pei Chen, Xue Li, Hongxiao Jia

**Affiliations:** a The National Clinical Research Center for Mental Disorders & Beijing Key Laboratory of Mental Disorders, Beijing Anding Hospital affiliated to Capital Medical University; b Department of Acupuncture and Moxibustion, Dongfang Hospital affiliated to Beijing University of Chinese Medicine, Beijing100078, China.

**Keywords:** acupuncture, functional magnetic resonance imaging, shift work disorder

## Abstract

**Introduction:**

Nearly 20% of night shift nurses will cause shift work disorder (SWD) due to the disruption of sleep-wake cycle, which not only affects the daily work efficiency, but also brings a huge burden on physical and mental health. Acupuncture is a safe and effective intervention on SWD. This trial will combine with functional magnetic resonance imaging (fMRI) to study the clinical effects and potential mechanism of acupuncture in the treatment of SWD.

**Methods and analysis:**

This is a randomized controlled neuroimaging trial, with enrolled participants, outcome assessors, and data statisticians blinded. 60 patients with SWD and 30 healthy controls who sleep regularly will be recruited and divided into divided into a control group, a true acupoints treatment group (TATG) and a sham acupoints treatment group (SATG) by the ratio of 1:1:1. The TATG and SATG will receive 8 sessions of acupuncture treatment in 4 weeks. Cognitive function scales and MRI scanning will be performed before and after 4-week acupuncture treatment. The control group will receive no intervention. Functional connectivity of intra-network and inter-network will be the primary outcome and effect indicator. The secondary outcomes included Repeatable Battery for the Assessment of Neuropsychological Status, Attentional Network Test, Pittsburgh Sleep Quality Index scale and needle sensation assessment scale. Neuroimage indicators will be correlated with clinical data and scores of cognitive function assessment to explore the possible mechanisms underlying the changes of brain activity caused by acupuncture treatment.

**Discussion:**

The results of this study will enable us to verify the therapeutic effect of acupuncture on SWD and explore the potential central mechanism of acupuncture on SWD from the change of brain activity.

## 1 Introduction

Shift work disorder (SWD) is a condition defined by excessive sleepiness or insomnia accompanied by total sleep time reduction.^[1]^ According to the epidemiology, SWD effects approximately 10% to 38% of the shift worker population. Nurses make up the largest proportion of shift workers (15%–20%).^[2]^ Evidence suggests that long-term shift work will not only affect work efficiency and patient satisfaction, but also lead to physical and mental health problems.^[3,4]^ In hence, given enough time, this may lead to more severe disorders, such as cardiovascular disease, cerebrovascular events, metabolic disorders, gastrointestinal complaints and multiple forms of cancer.^[5–7]^ Meanwhile, SWD also impairs cognition, such as attention and memory, which will directly affect work efficiency or even cause safety consequences not only for the individuals concerned, but also for society. Therefore, it is essential to explore an effective intervention on impairments of cognitive abilities for patients with SWD.

Medication is the main effective method to treat shift work sleep disorder,^[8]^ Such as benzodiazepines, nonbenzodiazepines and antihistamines. These drugs have a definite effect to a certain degree, while side effects may occur following a long time medication.^[9]^ The most common symptoms are headache, dizziness, nausea and anxiety.^[10]^ Clinical studies have confirmed that long -term taking benzodiazepines and non-benzodiazepines could impair attention, memory, response speed and mental activity,^[11,12]^ even increasing the risk of Alzheimer's disease.^[13]^ Furthermore, with the accumulation of drugs will lead to addiction.^[14]^ In hence, it is necessary to explore a safe and effective method to treat SWD.

Acupuncture, as one of the traditional treatment methods in China, has a long history in the treatment of sleep disorder.^[15]^ Abundant randomized controlled trials have confirmed that acupuncture treatment on insomnia has the advantages of stable effect and less side effects.^[16–19]^ A recent study also confirmed that manual acupuncture could improve memory function in chronic insomniacs. In hence, it is essential to definite efficacy of acupuncture treatment on cognitive decline. However, to our knowledge, there is no study focusing on acupuncture therapy on SWD.

In this study, we will conduct a randomized controlled trial to study the effect of acupuncture on cognitive abilities of SWD and reveal the underlying neuroimaging mechanism. The results will provide evidence and promote acupuncture therapy on SWD during clinical practice.

## 2 Methods

### 2.1 Study design

This is a randomized controlled, paralleled neuroimaging study, with patients, assessors and data statisticians blinded to the group assignment. A total of 60 patients with SWD and 30 healthy subjects will be recruited from October 2019 to December 2020 at Beijing Anding Hospital of Capital Medical University. They were divided into a control group, a true acupoints treatment group (TATG) and a sham acupoints treatment group (SATG) by the ratio of 1:1:1. The TATG and SATG will receive 8 sessions of acupuncture treatment in 4 weeks (2 sessions every week) by a trained acupuncture operator. Cognitive function scales and MRI scanning will be performed before and after 4-week acupuncture treatment. Meanwhile, the needle sensation will be evaluated by assessment scale after each session. The control group will receive no intervention.

**Figure 1:**
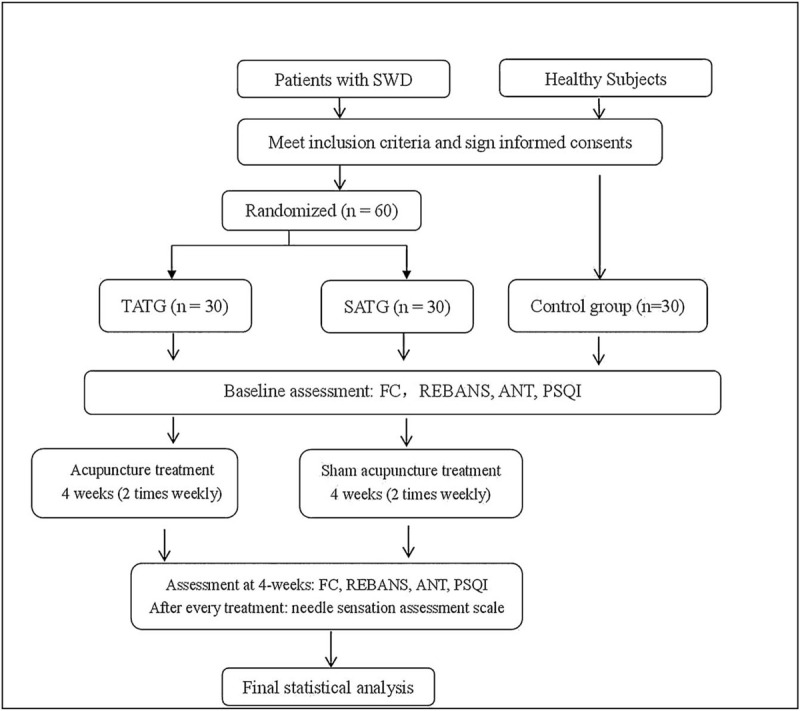
Flowchart of study design. ANT = Attentional Network Test, FC = functional connectivity, PSQI = Pittsburgh Sleep Quality Index scale, REBANS =  Repeatable Battery for the Assessment of Neuropsychological Status, SATG = sham acupoints treatment group, SWD = shift work disorder, TATG = true acupoints treatment group.

The study procedures are detailed in Figure 1.

### 2.2 Participants and recruitment strategy

A total of 90 participants will be recruited from October 2019 to December 2020 at Beijing Anding Hospital of Capital Medical University through posters and other publicity methods. Patients with SWD or healthy subjects who match the inclusion criteria will be considered potential participants. If they are eligible and interested in participating in the study, they will be fully informed of the entire study procedures, benefits and risks. Written informed consent will be obtained from the patient or their legally authorised representative before the allocation. Each participant has the right to withdraw from the study unconditionally.

### 2.3 Inclusion criteria of TATG and SATG

Patients will be enrolled if they meet the following criteria: meeting the diagnostic criteria for SWD according to International Classification of Sleep Disorders (2nd Edition) by the American Sleep Disorders Association;^[20]^ female nurses working at Beijing Anding Hospital; aged from 18 to 40 years, right-handed; continuous regular night shift for at least 1 year and work for 5 to10 years, at least 2 shift work per week; with no history of prophylactic or therapeutic medicine in the past 3 months; with no history of long-term use of analgesics.

### 2.4 Inclusion criteria of control group

Healthy subjects will be recruited following inclusion criteria: relative regularity of sleep in the past 12 months; aged 18 to 40 years, right-handed; sleeping less than 3 times per month after 23 o’clock in the latest 1 year and night shift less than 3 times a month in the latest 1 year.

### 2.5 Exclusion criteria

Participants will be ruled out if they meet any criteria below: pregnant or lactation; history of neurologic or psychiatric disorders; participating in such cognitive experiments within 1 year; any other health problems or poor physical conditions that may influence participation; any brain structure damage or abnormalites identified by MRI examinations; any history of alcohol or drug dependency; any MRI contraindications.

### 2.6 Sample size

When calculating the sample size during the acupuncture-neuroimaging study, the number in published literature and previous studies is often considered. On the one hand, in fMRI studies, millions of voxels are used to estimate the blood oxygen level dependent (BOLD) signal indirectly, and conventional power calculations often make no sense. On the other hand, the number of participants is often constrained by scanning time and costs. In hence, considering the published acupuncture-neuroimaging literature focusing on sleep disorder, the sample size range of 16 to 32 cases show enough statistical power for brain functional analysis.^[21]^ In the present study, we plan to recruit 60 patients with SWD and 30 healthy subjects with regular sleep.

### 2.7 Randomization and blinding

Randomization will be carried out in 2 steps. Firstly, according to the inclusion exclusion criteria, the 90 participants will be divided into experimental group and control group by the ratio of 2:1; secondly, the patient with SWD will be divided into TATG and SATG according to the computer-generated random sequence using Excel's rand function (1:1 ratio). The code will be sealed in opaque envelopes and opened by researchers not involved in the recruitment following informed consent procedures and baseline testing, with direct responsibility for treatment allocation. The researchers and acupuncturists will know the allocation. Meanwhile, the participants, outcome assessors and data analysts will be blinded.

### 2.8 Patient and public involvement

Participants in this study will not be involved in the design, recruitment or conduction of the study. After the MRI scanning, all participants will be informed of the results of neuroimaging data in the form of pictures.

## 3 Interventions

Patients with SWD will receive true or sham acupuncture treatments for 4 weeks, while healthy subjects will receive no intervention.

### 3.1 True group acupuncture

Patients in this group will receive 4-week manual acupuncture treatments at 10 verum acupoints (VA) (bilateral Fengchi (GB20), Benshen (GB15), Shenting (GV24), Shenmen (HT7), and Sanyinjiao (SP6)) with disposable sterile filiform needles (size 0.25 mm × 40 mm, Ande brand, manufactured by Ande Medical Appliance in Guiyang, Guizhou Province, China). Needles will be perpendicularly inserted into acupoints by clamping after skin disinfection using 75% alcohol. The depth of acupoint insertion was 0.5 to 1 inch, needles will be retained at the acupoints for 20 minutes after the deqi sensation is attained through the manipulation of lifting, thrusting and thrusting mild reinforcing-reducing method.

Two acupuncturist with over 5 years of clinical experience in acupuncture will be trained together and conduct the acupuncture treatments. After the first MRI scanning, the participants will receive a total of 8 sessions of acupuncture treatments in 4 weeks.

### 3.2 Sham acupuncture group

Patients in sham acupuncture group will receive 4-week manual acupuncture treatments at 10 sham acupoints (SA). According to the previous literature on setting locations of SA,^[22]^ the SA should be set aside with 1 inch beside the true point as the sham acupoint. In hence, the deqi experience of the participants in SATG will not be overemphasized. In spite of the locations of acupoints, the treatment procedures and course will be the same with TATG.

## 4 Measurements

**Figure 2:**
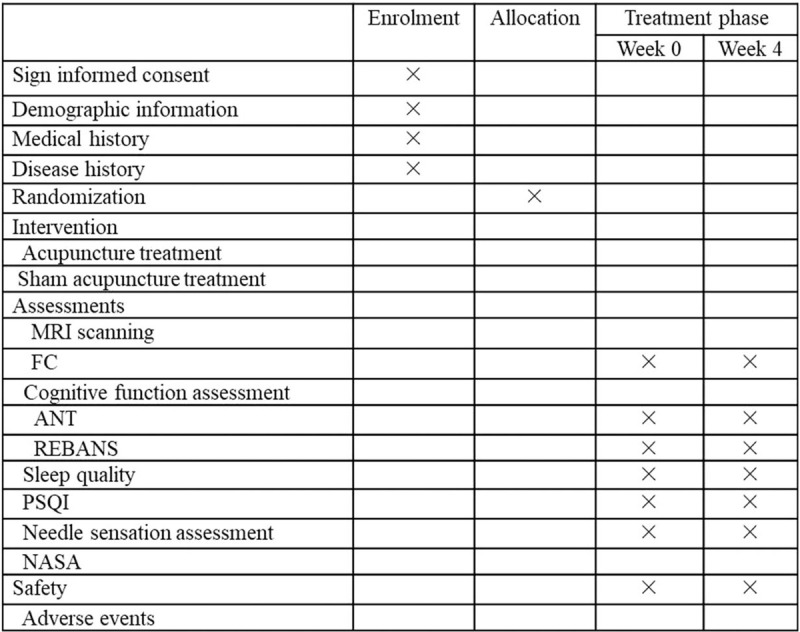
Schedule of study. ANT = Attentional Network Test, FC = functional connectivity, PSQI = Pittsburgh Sleep Quality Index scale, REBANS = Repeatable Battery for the Assessment of Neuropsychological Status.

The measurements consist of 3 parts including neuroimaging scanning assessments, cognitive function assessments, and needle sensation assessment. The study schedule is shown in Figure 2.

### 4.1 Demographic and basic clinical information collection

The demographic information (name, sex, age, height and weight), and the history of other concomitant diseases will be obtained at the baseline. Vital signs (blood pressure, pulse, respiration rate and temperature) will be measured before each scanning.

### 4.2 MRI scanning and neuroimaging outcomes

#### 4.2.1 MRI scanning protocol

MRI examinations, including resting-state blood oxygenation level-dependent imaging (RS-BOLD fMRI) and high-resolution anatomical T1-weighted imaging (T1W1), are designed for detecting cerebral function changes.

MRI Examination will be acquired applying a 3.0 Tesla MRI scanner (Siemens, Prisma Germany) at Anding Hospital, Beijing, China. Prior to scanning, all participants were asked to rest for 30 minutes before scanning. They were instructed to stay still, think of nothing in particular, keep eyes closed, and not to fall asleep during scanning. Earplugs were worn to attenuate scanner noise. The foam head holders were immobilized to minimize head movements during scanning.

Prior to the functional scanning, we collected high-resolution structural information for anatomical localization by using 3D MRI sequences. The resting-state fMRI data were collected using a single-shot, gradient-recalled echo-planar imaging sequence with the following parameters: repetition time = 2000 ms, echo time = 30 ms, flip angle = 90°, matrix = 64 × 64, field of view = 225 mm × 225 mm, slice thickness = 3.5 mm, gap = 1 mm, 32 interleaved axial slices, and 180 volumes.

#### 4.2.2 Neuroimaging outcomes

The neuroimaging outcomes will be acquired after the MRI scanning and analysis, mainly including functional connectivity (FC) within and between resting-state brain networks. FC will be taken as the primary outcome and effect indicator, which is an optimal parameter to reflect the functional connections within and between resting-state brain networks. Numerous studies revealed changes of the brain's intrinsic functional connectivity stemming from cognitive impairments on patients with sleep disorder.^[23,24]^ In hence, FC could reflect whether specific brain regions or resting-state brain networks are involved in improvements of cognitive functions after 4-week acupuncture treatments.

### 4.3 Cognitive function assessments and outcomes

The following cognitive function assessment scales, including Repeatable Battery for the Assessment of Neuropsychological Status (RBANS) and Attention Network Test (ANT), will be conducted at the baseline and after the 4-week acupuncture treatment by a trained assessor blinded to the random sequence. The results of RBANS and ANT will be taken as the secondary outcome in this trial.

#### 4.3.1 Repeatable Battery for the Assessment of Neuropsychological Status (RBANS)

RBANS, a cognitive screening test including 12 subtests, was utilized as the measure of global cognition. The RBANS generates 5 domain-specific index scores applied to evaluate 5 cognitive abilities: immediate and delayed memory, language, visuospatial/constructional ability and attention. In spite of delayed memory, a component based on 4 subtests, the other 4 are based on 2 subtests. The immediate memory index comprises the story memory and list learning subtests; the visuospatial/constructional index comprises the line orientation and figure copy subtests; the language index comprises of the photo naming and semantic fluency subtests. The attention index comprises the digit span and coding subtests; the delayed memory index comprises the list recall, story recall, list recognition and figure recall subtests. The Chinese version of the RBANS translated by Cheng et al was adopted in the current study.^[25]^ The test undergoes about 30 minutes. A trained neuropsychologist performed the test according to standardized procedures.

#### 4.3.2 Attention Network Test (ANT)

ANT, a cognitive task designed by Fan et al,^[26]^ was used to evaluate the efficiency of the alerting, orienting, and executive control. Subjects were required to click the mouse as quickly and accurately as possible to define the direction of the target, which was a rightward or leftward arrow in the center of screen and flanked on either side by 2 arrows in the same direction (congruent condition), or the opposite direction (incongruent condition). Each subject performed 6 blocks during this test, while each block last 5 minutes 42 seconds and included 36 trials plus 2 buffer trials. During each block, the 6 trial types were displayed in a predetermined counterbalanced order. All participants would be trained before the formal test. The entire test would be conducted on an experimental control computer.

### 4.4 Pittsburgh Sleep Quality Index scale (PSQI)

Pittsburgh Sleep Quality Index (PSQI), was used to evaluate the quality of sleep.^[27]^ This scale could measure the quality and patterns of sleep during the last 1 month. It included 7 domains: subjective sleep quality, use of sleep medication, sleep latency, sleep duration, habitual sleep efficiency, sleep disturbances and daytime dysfunction. Each items varied from 0 to 3 scale. An overall score of 5 or greater indicates poor sleep.

### 4.5 Needle sensation assessment

The needle sensation assessment scale will be conducted after each acupuncture treatment by the acupuncturists. The result may be a prognostic marker associated with the effectiveness of acupuncture and improvements of cognitive function, which will be taken as the additional outcomes.

“*Deqi*” is a needle response describing how the patients feel when they receive the needle insertion. The self-designed Needle Sensation Assessment Scale has been applied in previous studies,^[28]^ including “*Deqi*” features (tingling, numbness, sourness, aching and propagated feeling along the meridians) and rating of “*Deqi*”. Patients will describe the intensity of needle sensation by visual analogue scale (VAS) (0 stands for none, 10 stands for unbearable).

### 4.6 Adverse events

Any adverse events during the intervention period will be reported, such as bleeding, fainting, pain, infection or other severe events will be recorded with details such as the date of occurrence, degree and causality with the acupuncture treatment. If the situation worsens, the patients will be discontinued from the study and will be sent for further treatment.

### 4.7 Quality control, data collection and management

A series of training sessions will be held before the beginning of this study, which ensuring all researches get fully understood the standard procedures of this study. The medical imaging technicians will monitor the qualities of neuroimaging data after every scanning. The clinical and cognitive function data are required to be recorded on printed Case Report Forms (CRF) at each visit point. The completed CRFs will be undergone the double-entry verification in EpiData Entry software to ensure the accuracy of the data. All the MRI data will be stored in discs after finishing every scanning. After the data entry and verification as required, the CRF should be kept in the order of number, and filled with the search catalogue for reference. All original files shall be kept within the time limit specified.

## 5 Statistical analysis

### 5.1 Clinical data analysis

The measurement data of each visit in different groups will be described by means of mean ± standard deviation. Clinical data including scores of cognitive function assessments from the 3 groups will be compared using one-way ANOVA, and further statistical analysis will be conducted between 2 groups.

The counting data of each visit in different treatment groups were described by frequency (composition ratio). χ^2^ test or nonparametric test were used for the changes of the 3 groups before and after treatment.

### 5.2 Neuroimaging data preprocessing and analysis

#### 5.2.1 Resting-state fMRI data preprocessing

The fMRI data were preprocessed with Data Processing Assistant for Resting-Sate fMRI (DPARSF) software (http://rfmri.org/DPARSF). The first 10 volumes were discarded to allow the adaption of the subjects and the stabilization of the magnetization. The remaining volumes were slice-timing corrected for different acquisition in slice times and realigned to the first volume for head-motion correction. Based on the head motion data, the subjects were excluded according to the criteria of maximum translation as 1.5 mm and rotational parameters as 1.5 degrees in any direction. After that, the image data will be under the spatial normalization based on MNI space and resampled to 3 × 3 × 3 mm^3^. Finally, the data will be smoothed with a Gaussian kernel of 6 × 6 × 6 mm^3^ full width at half-maximum. Then, the data were temporally band-pass filtered (0.01–0.08 Hz) to reduce the low-frequency drift and high-frequency physiological noise.

#### 5.2.2 Functional connectivity analysis

Independent component analysis was applied to extract the resting-state brain networks by GIFT software (University of New Mexico, Albuquerque, NM). The number of independent components in all data was calculated by method of the minimum description length (MDL) technique. 30 components were estimated. Randlnit and Bootstrap operations were applied to evaluate the independent components. According to research objectives, independent components were selected based on the largest spatial correlation comparing with previous resting-state brain network templates. Then, resting-state brain networks were calculated by the way of goodness of-fit after removing the components not related to the spectrum of resting-state networks. The best goodness-of-fit score of components were normalized to *Z*-scores with Fisher's *r*-to-*z* transformation to acquire the entire brain *Z*-score map of each subject. Then we extracted time series of each network component to calculate the Pearson correlation coefficient (*r*) of each network component and other network time series, namely functional network connectivity (FNC). The resulting r values were normalized into *Z* values by Fisher-*Z* transformation. For group-level analyses, the functional connectivity of Intra-network and inter-network was performed by one-way ANOVA using SPM12 software (*P < *.05, corrected by false discovery rate (FDR) for multiple comparisons). Finally, BrainNet Viewer was used to display the result onto a 3D brain surface.

## 6 Discussion

Acupuncture is a common therapy for insomnia in China, and great progress has been made in the study of its pathogenesis.^[29]^ Systematic reviews and a large number of trials have confirmed that acupuncture is safe and effective in the treatment of insomnia.^[30,31]^ However, there are few studies on acupuncture treatment of SWD. Previous studies and literatures have shown that acupuncture can effectively treat insomnia and sleepiness of shift work disorder, and improve attention decline.^[32]^ In the current research, we will focus on the effect of acupuncture on cognitive functions of patients with SWD, and explore the neural mechanism of acupuncture on SWD through fMRI.

A large number of studies showed that, the activity of cerebral cortex and the internal and external functional connectivity of neural network were closely related to the occurrence of shift work disorder.^[33,34]^ Meanwhile, the fMRI technology provides a direct tool for the study of brain response to acupuncture intervention. Numerous studies revealed changes of the brain's intrinsic functional connectivity stemming from cognitive impairments on patients with sleep disorder.^[23,24]^ A previous study also reported that patients with SWD showed brain perfusion changes in multiple brain areas significantly correlated with insomnia severity.^[35]^ In this study, the relationship between acupuncture sensation and fMRI is preliminarily discussed. fMRI can infer that regional changes in brain activity reflect whether specific brain regions or resting-state brain networks are involved in improvements of cognitive functions after 4-week acupuncture treatments.

However, there are few studies on acupuncture in the treatment of SWD, which need to be further studied through larger, more rigorous and good randomized clinical trials. At the same time, the purpose of setting up the false acupuncture group in this study is to eliminate the placebo effect. However, it is difficult for Chinese patients to choose the sham acupoint as the acupuncture placebo. As patients may have their own acupuncture treatment experience and know the location of common acupoints. In hence, the blinding of patients in this study has a certain impact.

In conclusion, the purpose of this study is to evaluate effect of acupuncture on cognitive impairments of SWD and to interpret the neural mechanism of acupuncture on SWD through fMRI.

## Author contributions

YZN and XYL contributed equally in drafting this manuscript. YZN and XYL prepared the informed consent and finished trial registration. HY and XY are in charge of acupuncture treatment. PC is in charge of the motor assessment of participants. XYL and HY are in charge of screening and recruitment. YZN is in charge of neuroimage data processing and analysis. HXJ conceived and designed the study and is the corresponding author of the manuscript. All authors discussed, revised and approved of the final manuscript.
